# Cortical and Striatal Reward Processing in Parkinson’s Disease Psychosis

**DOI:** 10.3389/fneur.2017.00156

**Published:** 2017-04-24

**Authors:** Sara Garofalo, Azucena Justicia, Gonzalo Arrondo, Anna O. Ermakova, Pranathi Ramachandra, Carina Tudor-Sfetea, Trevor W. Robbins, Roger A. Barker, Paul C. Fletcher, Graham K. Murray

**Affiliations:** ^1^Department of Psychiatry, University of Cambridge, Cambridge, UK; ^2^Behavioural and Clinical Neuroscience Institute, University of Cambridge, Cambridge, UK; ^3^Cambridgeshire and Peterborough NHS Foundation Trust, Cambridge, UK; ^4^Department of Psychology, University of Cambridge, Cambridge, UK; ^5^Department of Clinical Neuroscience, University of Cambridge, Cambridge, UK; ^6^Cambridge University Hospitals NHS Foundation Trust, Cambridge, UK

**Keywords:** ventral striatum, posterior cingulate cortex, prediction error, reinforcement learning, fMRI

## Abstract

Psychotic symptoms frequently occur in Parkinson’s disease (PD), but their pathophysiology is poorly understood. According to the National Institute of Health RDoc programme, the pathophysiological basis of neuropsychiatric symptoms may be better understood in terms of dysfunction of underlying domains of neurocognition in a trans-diagnostic fashion. Abnormal cortico-striatal reward processing has been proposed as a key domain contributing to the pathogenesis of psychotic symptoms in schizophrenia. This theory has received empirical support in the study of schizophrenia spectrum disorders and preclinical models of psychosis, but has not been tested in the psychosis associated with PD. We, therefore, investigated brain responses associated with reward expectation and prediction error signaling during reinforcement learning in PD-associated psychosis. An instrumental learning task with monetary gains and losses was conducted during an fMRI study in PD patients with (*n* = 12), or without (*n* = 17), a history of psychotic symptoms, along with a sample of healthy controls (*n* = 24). We conducted region of interest analyses in the ventral striatum (VS), ventromedial prefrontal and posterior cingulate cortices, and whole-brain analyses. There was reduced activation in PD patients with a history of psychosis, compared to those without, in the posterior cingulate cortex and the VS during reward anticipation (*p* < 0.05 small volume corrected). The results suggest that cortical and striatal abnormalities in reward processing, a putative pathophysiological mechanism of psychosis in schizophrenia, may also contribute to the pathogenesis of psychotic symptoms in PD. The finding of posterior cingulate dysfunction is in keeping with prior results highlighting cortical dysfunction in the pathogenesis of PD psychosis.

## Introduction

Psychotic symptoms, such as hallucinations, paranoia, and delusions, are present in about 15–40% of Parkinson’s disease (PD) patients and may have a major impact on quality of life and likelihood of nursing home placement (1–5).

Several pathophysiological mechanisms have been proposed to explain how psychotic symptoms develop in PD. It has been suggested that visual hallucinations in PD develop as a consequence of cortical degeneration in posterior cortical areas (6, 7). An alternative to a single locus account of PD psychosis (PDP) is that the psychosis emerges through interactions between subcortical and cortical pathological processes, or through the interaction of frontal and posterior cortical deficits, or through interaction between intrinsic disease-related factors and extrinsic drug factors (3, 4, 7–11).

There have been comparatively few attempts to integrate mechanistic explanations of psychotic symptoms in PD with current models of their pathophysiology in primary psychiatric illness. In spite of clear potential differences in the pathogenesis of psychiatric symptoms in PD compared to primary psychiatric illness (2, 12), there may be common pathways for the manifestation of certain neuropsychiatric symptoms across traditional diagnostic categories (6, 13). A longstanding candidate contributor cause of psychosis in PD is a dopaminergic system perturbed by a combination of intrinsic pathology and extrinsic medication, as dopaminergic drugs are strong risk factors for the manifestation of psychotic symptoms in PD: odds ratios for hallucinations are up to fivefold for dopamine agonist treatment compared to placebo in a meta-analysis (14). Furthermore, withdrawal of dopaminergic medication or dose reduction frequently brings relief from psychotic symptoms (11). There is already a large literature implicating dopamine and serotonin neurotransmission in the pathogenesis and treatment of schizophrenia spectrum psychosis and preliminary evidence linking fronto-striatal dopaminergic function to schizotypal traits in the general population (15–18). The recently identified role for serotonin in the pathology and treatment of PDP (19, 20) further weight for consideration of at least some potential commonalities in the pathophysiology of psychosis in schizophrenia spectrum conditions and PDP.

Abnormal dopaminergic (21, 22) and cortico-striatal (23, 24) function manifesting in altered processing of rewards and reward prediction, have been proposed as key factors contributing to the pathogenesis of psychotic symptoms. These accounts have received empirical support in the study of psychosis in schizophrenia spectrum disorders and preclinical models of psychosis (23, 25–31), but have not been tested in PDP. However, there is already evidence for this from a number of studies of both cortical and subcortical dysfunction during reward processing in PD (32–34). These factors merit the further investigation of cortico-striatal reward processing as a putative neurocognitive process potentially contributing to the pathogenesis of PDP.

In this study, we have focused on the neural basis of prediction of a reward and the representation of reward prediction error: processes with previous theoretical and empirical support for their involvement in the pathophysiology of psychotic symptoms in schizophrenia. A recent meta-analysis found evidence of ventral striatal dysfunction associated with both of these neurocognitive processes in schizophrenia spectrum psychosis (31), so we elected to study the ventral striatum (VS) as a region of interest (ROI) in our cohorts of PD patients. We aimed to complement this subcortical ROI with analysis within two cortical ROIs, the ventromedial prefrontal cortex and posterior cingulate cortex (PCC), where we previously identified a link between abnormal activation during reward processing and psychotic symptom expression (23).

Our hypothesis was that patients with PD psychosis should present reductions in PCC, ventromedial prefrontal cortex, and ventral striatal activations associated with reward anticipation and prediction error relative to PD patients without psychosis.

## Materials and Methods

### Participants

The final sample was composed of 53 participants: 17 patients with a diagnosis of PD without any psychotic symptoms (PD); 12 patients with a diagnosis of PD and a history of current or previous psychotic symptoms (lifetime CAARMS scoring equal to or greater than 3 in global and frequency scales) (PDP); 24 healthy volunteers, with no history of neurological, psychiatric, or medical disorders (controls). The initial sample was composed of 66 participants. Ten participants (8 PD, 1 PDP, 1 control) were excluded due to technical programming problems, which occurred during the task (responses not recorded, *N* = 9), or to the fMRI scanning (wrong scanning protocol, *N* = 1). Three additional subjects (2 PD, 1 control) were excluded because they did not complete the task. Patients were recruited *via* the PD research clinic at the John van Geest Centre for Brain Repair (VGB); all fulfilled the Queen Square Brain Bank Criteria for idiopathic PD (35) and remained on their usual medications during testing. Each patient’s dopaminergic drug regime was converted to an equivalent l-DOPA dose (36). Patients with dementia were excluded [operationalized as a Mini-Mental State Examination (37) score less than 24]. Descriptive statistics of the sample are reported in Table 1. All subjects had normal or corrected-to-normal visual acuity and were without any contraindications for MRI scanning.

**Table 1:** **Descriptive statistics**.

Characteristics	Control	PD	PD psychosis	Test	*p*-Value
**Demographics**					
Participants, *n*	24	17	12		
Age, mean (SD), years	61.91 (5.83)	63.29 (9.94)	60.83 (6.6)	*F*(2,50) = 0.39	0.68
Gender, M/F (% male)	10/14 (43)	10/7 (56)	6/6 (50)	χ^2^ (2) = 1.17	0.55
Handedness R/L (% right)	22/1 (96)	15/3 (83)	11/1 (92)	χ^2^ (2) = 0.15	0.92
White-British, *n* (%)	22 (91.66)	17 (100)	12 (100)	χ^2^ (2) = 2.51	0.28
Educational qualifications					
No qualifications (%)	3 (12.5)	1 (5.88)	0 (0)	χ^2^ (2) = 1.89	0.38
16/18 years old qualif. (%) (GSCSEs, A-level or eq.)	10 (41.66)	6 (35.29)	6 (50)	χ^2^ (2) = 0.62	0.73
Degrees, advanced vocational qualif. (%)	11 (48.83)	10 (58.82)	6 (50)	χ^2^ (2) = 0.67	0.71
**Cognitive function**					
MMSE—total, mean (SD)	30.30 (3.08)	28.94 (1.59)	28.41 (1.37)	*F*(2,49) = 3.07	0.07
Estimated IQ, mean (SD)	102.04 (11.92)	89.64 (14.59)	86.54 (13.45)	*F*(2,49) = 7.1	0.002
	Controls vs all PD *p* < 0.01; PD vs PDP *p* = 0.1				
**PD**					
Hoehn and Yahr stage, % 1/2/3/4/5	–	59/35/0/0/6	60/10/30/0/0	χ^2^ (1) = 0.5	0.72
Duration, years mean (SD)	–	10.94 (10.38)	16.42 (28.77)	*t*(27) = −0.81	0.43
**Medications**					
Levodopa equivalent dosage, mean (SD)	–	614.81 (503.42)	714.03 (531.42)	*t*(22) = 0.5	0.61
Levodopa therapy, *n* (% yes)		13 (76.47)	9 (75)	χ^2^ (1) = 0.00	0.9
DA agonist, *n* (% yes)		8 (47.05)	10 (83.33)	χ^2^ (1) = 2.54	0.11
Antidepressants, *n* (% yes)	–	7 (41.17)	1 (8.33)	χ^2^ (1) = 2.33	0.12
Anxiolytics, *n* (% yes)	–	3 (17.64)	3 (25)	χ^2^ (1) = 0.009	0.92
**Psychopathology**					
BDI total, mean (SD)	3.08 (3.69)	8.88 (4.94)	12.66 (7.83)	*F*(2,50) = 14.73	<0.001
	Controls vs all PD *p* < 0.003; PD vs PDP *p* = 0.1				
Apathy Evaluation Scale total, mean (SD)	–	32.1 (10.7)	33 (8.6)	*t*(22) = 0.23	0.82
**CAARMS score equal or over 3, global rating scales**					
Unusual thought content	–	–	0	–	–
Non-bizarre ideas, *n* (%)	–	–	3 (25)	–	–
Perceptual abnorm., *n* (%)	–	–	9 (75)	–	–
Disorganized speech, *n* (%)	–	–	2 (16.66)	–	–

*Characteristics of the whole sample separated into the three groups: controls, Parkinson’s disease (PD) without, and PD with psychosis. The inclusion criterion for the psychosis group is Lifetime Comprehensive Assessment of At Risk Mental States scale (CAARMS, which measures psychotic symptoms) scoring equal or over 3 in global and frequency scales. BDI, Beck Depression Inventory. MMSE, Mini-Mental State Examination. IQ was estimated using the Culture Fair test. Only one patient in the study (PDP group) was taking antipsychotic medication (100 mg quetiapine twice daily)*.

### Operational Definition of PDP

We defined the presence of PDP using a sensitive instrument measuring mild psychotic symptoms from the field of early detection of schizophrenia spectrum psychosis: the Comprehensive Assessment of At Risk Mental States (38, 39). This scale allows for sensitive measurement of a spectrum of psychotic experiences and includes measures on both the frequency and severity of symptoms. For example, the perceptual aberration scale scores of severity can range from 0 (no abnormality), 1 (questionable perceptual changes), 2 (heightened or dulled perceptions, illusions), through to 6 (a true hallucination that the patient believes true at the time and after the experience). The instrument takes these measures and operationalizes a categorical definition of attenuated psychosis (e.g., on the perceptual abnormalities scale, a 3 would be required for severity and frequency, indicating at least an intense illusion, “subject unsure of the nature of the experience,” with frequency of at least once per month if lasting over an hour per occasion or over 3 times per week if shorter). We reasoned that this threshold has been meaningful in clinical psychiatric practice (40) and could represent not only a sensitive but also a clinically meaningful way of classifying PD patients as having experienced psychosis or not. As we were interested in the propensity to psychosis in PD, we included patients in the PDP group with current and past (post PD onset) psychosis, even if the psychotic symptoms had resolved or improved by the time of the study.

### Rating Scales

The Beck Depression Inventory (41) was used to assess depressive symptoms during the last 2 weeks. The Apathy Evaluation Scale (self-complete) assessed apathy (42). IQ was estimated using the Culture Fair test (43). The Hoehn and Yahr scale (44) was used to assess the stage of PD in patients.

### fMRI Cognitive Task

During the fMRI scan, subjects performed a computerized instrumental learning task based on that used in previous studies (23, 45–48).

**Figure 1:**
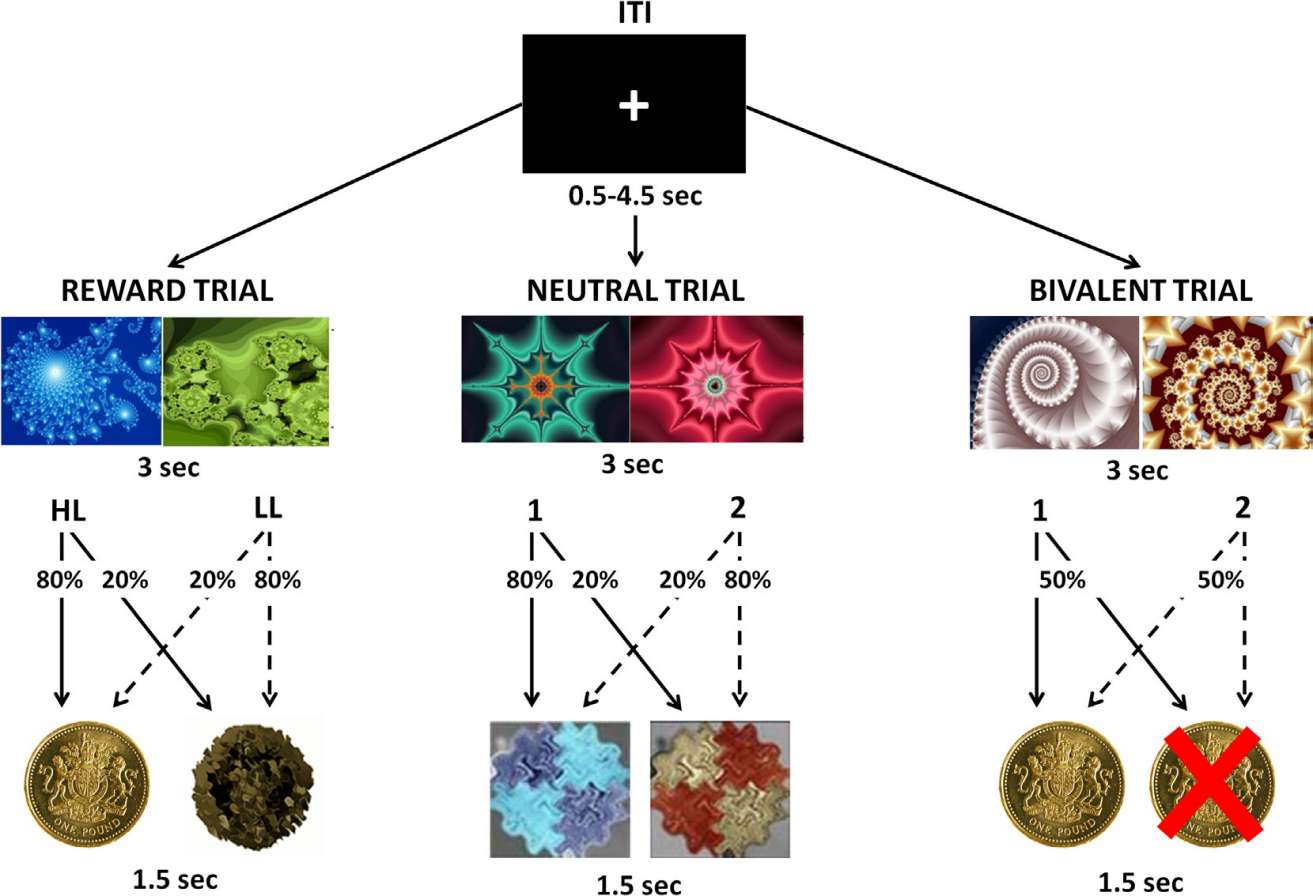
**Visual representation of the instrumental conditioning task**. Participants were presented with two fractal images and were required to choose one of them in order to maximize their payoffs. Each choice was followed by a visual feedback indicating the associated outcome. During Reward Trials, one choice was associated with an 80% chance to win £1 [High-Likelihood (HL)] and the other choice was associated with a 20% chance to win £1 [Low-Likelihood (LL)]. During Bivalent Trials, each choice was associated with a 50% chance of either losing or winning £1. During Neutral Trials, each choice was associated with an 80/20% chance of receiving two kinds of neutral feedbacks.

During the task, participants were presented with two fractal images and required to choose one of them, with a button press, in order to maximize their payoffs. Each choice was followed by a visual feedback indicating the associated outcome. There were three possible trials: during Reward Trials, one choice was associated with a £1 win in 80% of trials and with neutral feedback in 20% of trials [High-Likelihood (HL)], whereas the other choice was associated with a neutral outcome in 80% of trials and with a £1 win in 20% of trials [Low-Likelihood (LL)]; during Bivalent Trials, each choice was associated with a 50% chance of either losing or winning £1; during Neutral Trials, each choice was associated with a 80/20% chance of receiving two kinds of neutral feedback (Figure 1). Each trial type was repeated 30 times in a pseudo-random sequence, for a total of 90 trials. The order and position of the pictures presented were counterbalanced across trials of the same kind. To win money, the participants had to learn, by trial and error, to select the stimulus that was more likely to produce a reward. All participants were informed that the total amount won during the task would be paid to them at the end of the experimental session. All participants underwent a training session before entering the scan in which they received an explanation of the task, and practiced it for 5 min.

### fMRI Data Acquisition and Analysis

A Siemens Magnetom Trio Tim operating at 3 T was used to collect imaging data. Gradient-echo, echo planar T2*-weighted images, depicting *bold* contrast were acquired from 35 non-contiguous oblique axial planes to minimize signal drop-out in ventral regions. TR was 1,620 ms; echo time was 30 ms; flip angle was 65°; in-plane resolution was 3.0 × 3.0, matrix size was 64 × 64; field of view was 192 mm × 192 mm, bandwidth 2,442 Hz/Px. A total of 550 volumes per subject were acquired (27 slices each of 2 mm thickness, interslice gap 1 mm). The first five volumes were discarded to allow for T1 equilibration effects. Slice-timing correction was applied.

The data were analyzed in SPM 12 software (Wellcome Department of Cognitive Neurology, London, UK). Images were realigned, spatially normalized to a standard template, and spatially smoothed with a Gaussian kernel (8 mm at full-width half-maximum). A high-pass filter was applied (128 Hz).

A within subject analysis (often referred to as “first-level” analysis) was undertaken, regressors (explanatory variables) used in the general linear model were as follows: bivalent cue presentation; neutral cue presentation; reward cue presentation; loss feedback during Bivalent Trials; win feedback during Bivalent Trials; neutral feedback 1 during Neutral Trials; neutral feedback 2 during Neutral Trials; neutral feedback during Reward Trials; and win feedback during Reward Trials. All regressors were modeled as 0 second events and convolved with the SPM canonical hemodynamic response function. Motion parameters were used as additional regressors to control for movement.

Contrasts of interest were computed at the individual subject level and then taken to a group level for statistical analysis. There were two main contrasts of interest: reward anticipation and prediction error. To measure reward anticipation, a contrast between the presentation of the reward cue minus the neutral cue was performed. To examine prediction error, a contrast between the presentation of win feedbacks during bivalent trials (where a reward is unexpected) minus a win on Reward Trials (where a win is expected) was performed.

The main contrast of interest at the group level was PDP in comparison to PD without psychosis, as this comparison controls for many of the potential confounds that would be needed in any comparison of PDP with controls. We focused on *a priori*-defined ROIs comprising medial orbitofrontal cortex, PCC, and bilateral VS (see Figure S1 in Supplementary Material). A single mask from these regions was created using spheres centered on the following MNI coordinates taken from the meta-analysis of reward anticipation by Liu et al. (49): 0, −30, 32 (posterior cingulate, 12 mm diameter); 0, 34, −8 (ventromedial prefrontal cortex, 8 mm diameter); and bilateral VS (12, 10, −4, 8 mm diameter, and −12, 10, −4, 8 mm diameter). Results were considered significant where peak voxel activation survived a family-wise error small volume correction across the voxels of this mask at *p* < 0.05. For display of the clusters that passed this small volume correction and calculation of their cluster size, the uncorrected threshold was *p* = 0.005.

### Movement

To ensure that the three groups did not differ in the amount of movement during the fMRI scanning session, the averaged time series of translations and rotations along the three axes (*x, y, z*), as estimated during the realignment phase of pre-processing, were compared across groups.

Two separate mixed-effects models were used, with Group (controls/PD/PDP) as the independent variable and translations (millimeter) or rotations (degrees) as the dependent variable.

No group differences were found either in the amount of translations (*p* = 0.35; part. η^2^ = 0.04) or rotations (*p* = 0.32; part. η^2^ = 0.04).

### Behavioral Analysis

**Figure 2:**
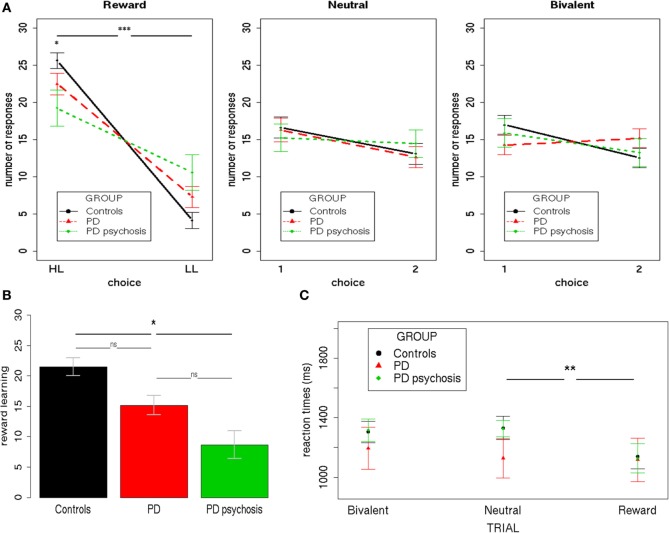
**Behavioral performance**. **(A)** The total number of responses during the three trial types (Reward/Neutral/Bivalent) for the three groups (controls/PD/PDP). **(B)** The reward learning index (HL–LL) for the three groups (controls/PD/Parkinson’s disease with psychosis). **(C)** Mean reaction times during the three trial types (Reward/Neutral/Bivalent) for the three groups (controls/PD/Parkinson’s disease with psychosis). HL, high-likelihood; LL, low-likelihood; 1, choice one; 2, choice two; PD, Parkinson’s disease. Bars indicate SE (***p < 0.0001; **p < 0.001; *p < 0.05).

To analyze participants’ performance during the task, three separate 3 × 2 mixed-effects models were performed for each trial condition (Reward/Neutral/Bivalent), using the total number of responses as the dependent variable (Figure 2A). Group (controls/PD/PDP) and Choice (HL/LL or 1/2) were used as the independent variables.

### Demographic and Clinical Analysis

Analyses of the demographic and clinical characteristics from the whole sample are reported in Table 1. χ^2^ was used to compare frequencies. For comparing means between groups, *t*-tests (comparison between PD/PDP) or ANOVAs (comparison between controls/PD/PDP) were used as appropriate. Significant differences were further investigated *via* Bonferroni-corrected *post hoc* analysis.

## Results

### Behavioral Results

Results from Reward Trials showed a significant main effect of Choice [*F*(1,50) = 83.22; two-tailed *p* < 0.001; part. η^2^ = 0.62], evidencing a higher number of HL over LL choices and a Group X Choice interaction [*F*(2,50) = 4,42; two-tailed *p* = 0.02; part. η^2^ = 0.15]. Bonferroni-corrected *post hoc* tests revealed a significant difference only between controls and PDP for both HL (*p* < 0.01) and LL (*p* < 0.01) choices. On average, HL choices were performed more by controls (mean = 25.62, SD = 5.21) than PDP (mean = 19.25, SD = 8.31); whereas LL choices were performed less by controls (mean = 4.12, SD = 5.31) than PDP (mean = 10.58, SD = 8.36).

Results from Neutral and Bivalent Trials showed no statistically significant effect (*p*’s > 0.1).

A further 3 × 3 mixed-effects model was performed using the reaction times as the dependent variable (Figure 2C). Group (controls/PD/PDP) and Trial type (Reward/Neutral/Bivalent) were used as the independent variables. Results showed a significant main effect of Trial type [*F*(1,87) = 5.09; two-tailed *p* = 0.007; part. η^2^ = 0.03]. Bonferroni-corrected *post hoc* tests revealed a significant difference only between Reward and Neutral Trials (*p* = 0.006). All other effects were not significant (*p*’s > 0.1).

Overall, these results indicate that participants, irrespective of the group, showed contingency learning during Reward Trials, expressing both as a preference for the HL choice (i.e., more frequently associated with a reward—80%) over the LL choice (i.e., less frequently associated with a reward—20%) and at faster reaction times when choosing during such trials, as compared with Bivalent and Neutral Trials—a well-replicated finding that has been referred to as “reinforcement-related speeding” (29, 45, 50). Nevertheless, controls presented a significantly higher ability to distinguish between choices with a higher and lower chance of rewards, as compared with PD patients with psychosis. Such a difference was subsequently directly tested by creating an index of the ability to learn from rewards (reward learning), obtained by subtracting for each subject the number of LL responses from the number of HL responses (Figure 2B). A mixed-effects model was performed using this reward learning index as the dependent variable and Group (controls/PD/PDP) as the independent variable. Results showed a significant main effect of Group [*F*(2,50) = 4.37; two-tailed *p* = 0.02; part. η^2^ = 0.15]. This further analysis confirmed what had been observed with the previous analysis: control participants were better able to differentiate between choices with higher and lower chance of reward and to use this information to adapt their behavior. In this regard, it is important to note that PD patients with psychosis still showed a significant difference between HL and LL choices (HL > LL), so they did learn the contingencies during Reward Trials. Critically, no significant difference between PD patients with and without psychosis was found.

Neither choice preferences nor differences in reaction times were evidenced in Neutral and Bivalent Trials.

We also examined the number of actual rewards received during Reward Trials. A mixed-effects model was performed using the number of rewards received as the dependent variable and Group (controls/PD/PDP) as the independent variable. Results showed a significant main effect of Group [*F*(2,50) = 5.11; two-tailed *p* = 0.01; part. η^2^ = 0.17]. Bonferroni-corrected *post hoc* tests revealed a significant difference only between controls and PDP (*p* < 0.02). On average, more rewards were received by controls (mean = 21.16, SD = 0.22) relative to PDP (mean = 17.25, SD = 4.39). Critically, no significant difference between PDP (mean = 17.25, SD = 4.39) and PD (mean = 18.17, SD = 4.39) was found (*p* > 0.9).

### Imaging Results

#### Reward Anticipation

**Figure 3:**
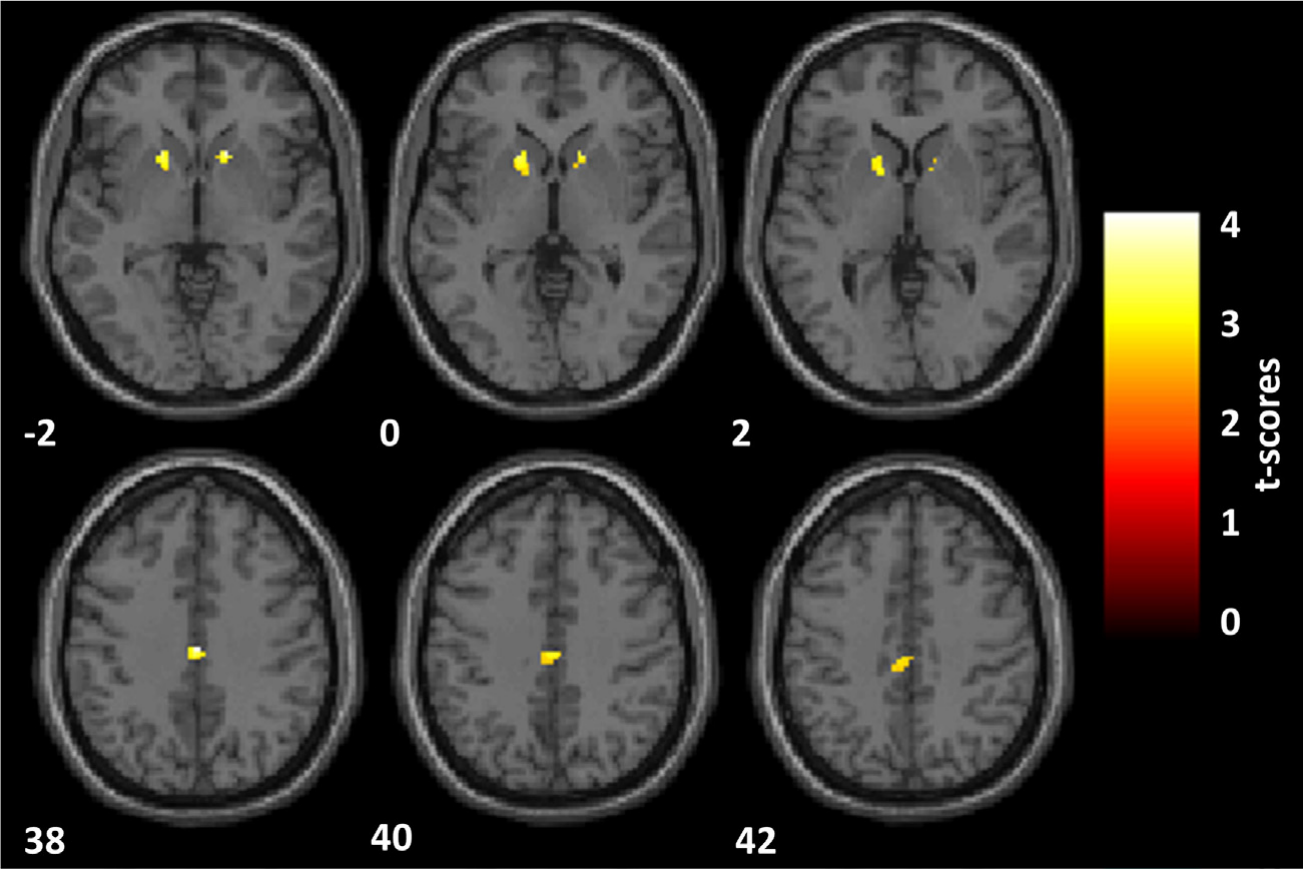
**Reward anticipation**. Statistical parametric maps of the reward anticipation calculated on the contrast of reward minus neutral cues onset. Analyses are restricted to the regions of interest and overlaid on a standard space structural image. Significant effects, thresholded at *p* = 0.005 and family-wise error corrected at *p* = 0.05, are shown in yellow. The left hemisphere is displayed on the left. MNI coordinates are reported. Parkinson’s disease patients with psychosis, as compared with Parkinson’s disease patients, showed a significantly reduced activation within the posterior cingulate cortex and the right ventral striatum (VS) and a marginally significant reduced activation in the left VS.

PD psychosis patients, as compared with PD patients, reported a significantly reduced activation in two clusters (Figure 3): one located within the PCC sphere [peak MNI coordinates *x, y, z* = 2, −20, 38; peak *z* = 3.65; peak *p* = 0.031 (small volume corrected); voxels = 59] and one located in the right VS [peak MNI coordinates *x, y, z* = 16, 16, −2; peak *z* = 3.60; peak *p* = 0.037 (small volume corrected); voxels = 21]. A third cluster located in the left VS showed marginally significant reduced activation in PDP as compared to PD patients [peak MNI coordinates *x, y, z* = −16, 16, −2; peak *z* = 3.03; peak *p* = 0.077 (small volume corrected); voxels = 44].

**Figure 4:**
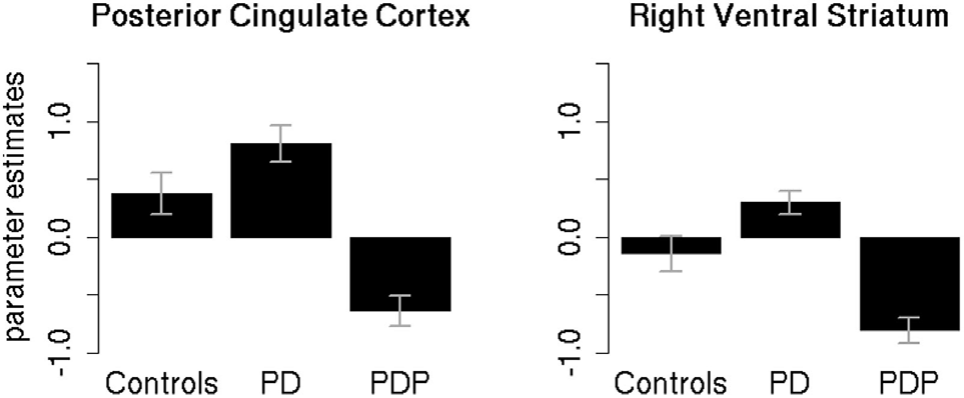
**Mean parameter estimates in the significant clusters**. Mean parameter estimates of the reward anticipation contrast, extracted for the three groups (controls, PD, PDP), from the significantly different clusters found within the posterior cingulate cortex and right ventral striatum regions of interest. PD, Parkinson’s disease patients; PDP, Parkinson’s disease patients with psychosis. Bars represent SE.

Parameter estimates of the two significantly different clusters (PCC and right VS) were extracted for the three groups (controls, PD, PDP) and statistically compared (Figure 4).

Two separate mixed-effects models, one for the PCC and one for the right VS, were performed using the extracted parameter estimates as the dependent variable. Group (controls/PD/PDP) was used as the independent variable. As the clusters are defined by differences between the PD and PDP groups, it is to be expected that these groups will show differences, but extraction of the cluster scores allows visualization of results and comparison against the healthy control group without PD.

Results in the PCC cluster showed a significant main effect of Group [*F*(2,50) = 4.89; two-tailed *p* = 0.01; part. η^2^ = 0.16]. Bonferroni-corrected *post hoc* tests revealed a significant difference between PD and PDP groups (*p* = 0.009). All other effects were not significant (*p*’s > 0.2).

Results on the right VS cluster showed a significant main effect of Group [*F*(2,50) = 5.54; two-tailed *p* = 0.007; part. η^2^ = 0.18]. Bonferroni-corrected *post hoc* tests revealed a significant difference between PD and PDP (*p* = 0.005) and a marginally significant difference between controls and PDP (*p* = 0.058). Controls and PD were not significantly different (*p* = 0.73).

There were no differences that survived correction for multiple comparison between PDP and PD groups on the whole-brain analysis.

Although the PD and PDP groups did not significantly differ on depressive symptoms, nevertheless, the PDP group had non-significantly more depressive symptoms than the PD group. We, therefore, compared the extracted parameter estimates between PDP and PD, controlling for depressive symptoms using ANCOVA: the difference between PDP and PD remained in the PCC cluster [*F*(2,26) = 10.35, *p* = 0.003] and in the right VS [*F*(2,26) = 12.92, *p* = 0.001].

#### Prediction Error

There were no differences that survived correction for multiple comparisons between PDP and PD groups on ROI or whole-brain analysis.

## Discussion

In this study, we found abnormal brain activation in PDP associated with reward anticipation in the VS and PCC. The finding of psychosis-related abnormal striatal activity is novel for PD but in line with a significant body of literature in schizophrenia spectrum psychosis showing—across a wide range of procedures and manipulations—dysfunctional striatal processing of rewards (29, 51–56). Indeed, Radua et al. (31) recently confirmed on meta-analysis the presence of ventral striatal dysfunction associated with reward anticipation in schizophrenia spectrum psychosis.

We found a significant deficit in reward anticipation activation in the right VS in the PDP group, but only a marginally significant deficit in the left VS. This differential sensitivity might reflect important biological processes or could simply be due to chance. A recent neurofunctional meta-analysis by Radua et al. (31) directly investigated a possible VS lateralization of reward processing in psychosis. The authors analyzed 23 studies (917 patients) for reward anticipation, 9 studies (358 patients) for reward feedback, and 8 studies (314 patients) for reward prediction error, reporting no differences between left and right VS in any of the reward processes considered. Based on these results, we feel it would be premature to draw conclusions from slightly stronger group differences for the right VS found in the present study.

The functional neuroanatomical basis of psychotic symptoms in PD has received little study using fMRI. The present study is the first cognitive fMRI study, to our knowledge, to compare PD patients with and without a history of psychotic symptoms considered broadly as we have done here (although it is probable that some of the previous studies of hallucinations included patients with delusional ideation by chance). Previous studies have focused on comparing brain activation between PD patients with and without hallucinations, but only a handful have been conducted (57–61). Some studies did not make corrections for multiple comparisons, and one had a sample size of only three patients with hallucinations. Meppelink et al. (59) examined visual processing in PD patients with and without hallucinations and found robust evidence for visual processing deficits in the patients with hallucinations in the superior frontal, lingual, and fusiform gyri. There were additional areas of group difference that did not survive correction for multiple comparisons.

A number of related theories have posited that abnormal brain mechanisms of learning and predicting the environment could contribute to the pathogenesis of psychosis (21, 26, 28, 62). The striatum is key in learning and updating ones beliefs in the light of experience (63); hence striatal dysfunction is a plausible candidate process that could underpin the formation of abnormal beliefs (64). Contemporary neuroscience accounts of brain predictive processing emphasize the importance of prediction in determining perceptual experience, potentially explaining how brain predictive dysfunction could lead, not just to delusions, but also hallucinations (65). We previously showed that the degree of disruption to the representation of reward expected value in the PCC secondary to methamphetamine administration predicted the severity of psychotic symptoms induced by the drug (23); that previous study is consistent with the current results implicating posterior cingulate reward anticipation dysfunction in the pathophysiology of psychotic symptoms.

Ramírez-Ruiz et al. (60) examined activations elicited by a visual attention (face recognition) task. They found, using a cluster-correction threshold, reduced frontal activation in the superior and inferior frontal cortex in hallucinating patients compared to controls. Ramirez-Ruiz argued that their findings of frontal deficits in association with hallucination might indicate, in part, a particular difficulty in patients prone to visual hallucinations in differentiating between relevant and irrelevant visual information. A failure to differentiate between motivationally salient and irrelevant information has been argued to be a key mechanism in the pathophysiology of psychotic symptoms in schizophrenia (62). Our findings of reduced ventral striatal and PCC activation in the comparison of rewarding and neutral cues are interpretable as impairment in differential anticipation in relation to motivational salient and irrelevant information. Yao and colleagues’ (66) examination of resting-state fMRI differences between 12 PD patients with, and 12 without, visual hallucinations found resting-state differences in posterior cingulate connectivity between the groups, with no evidence of cortical thickness differences. Although we failed to find evidence of frontal reward prediction dysfunction in PDP or of prediction error-associated dysfunction in any of ROIs, this absence of evidence does not necessarily exclude these processes and regions being implicated in the pathogenesis of PDP.

Analysis of the behavioral performance in our study indicated that participants acquired a preference for the highly rewarded choice during Reward Trials (HL > LL), thus showing learning of the task contingencies. PD patients with psychosis displayed a significantly lower preference for the HL choice relative to controls. Nevertheless, in the PDP group, there was a significant preference for HL over LL choices, thus demonstrating learning. Critically, there were no differences between patients with and without psychosis, indicating that the imaging results were not confounded by learning deficits in PDP.

Although we cannot draw direct inferences about the neurochemical basis of our findings, it is possible that monoaminergic function may contribute to our findings. There is evidence that dopamine abnormalities in the striatum, and possibly also serotoninergic abnormalities, contribute to psychotic symptoms in schizophrenia (15, 17), and there are separate lines of evidence suggesting that dopaminergic and serotonergic factors may contribute to psychosis in PD (11, 19, 20). Previous studies have indicated that serotoninergic manipulations modulate fMRI activation and connectivity in the PCC (67, 68). Serotonin neurons have been shown to be important in representing reward cues and outcomes (69), and serotonin receptors, including 5HT2a, are widely expressed in the cortex, including in the posterior cingulate (70, 71). 5HT2a receptors have also been associated with more general cognitive flexibility (72–74), which could also be relevant for the generation of psychotic symptoms; one recent research study found evidence that that a failure to flexibly adapt learning to the degree of volatility in the environment could contribute to the pathogenesis of psychosis (75). Subcortical dopaminergic neurotransmission plays a key role in reward processing and motivation (76, 77), and there is also evidence for monoaminergic modulation of reward predicting activity even in posterior cortical regions (78).

Our study has a number of strength and limitations. A novel aspect of the study is the attempt to reconcile investigative methods (interview scales, fMRI paradigm) examining the physiology of psychosis in PD and schizophrenia spectrum conditions. The small number of patients with PDP is common in this research field but remains a limitation, and definitive conclusions will require a much larger sample size. Another limitation is that we made no attempt to characterize the neuropsychological performance of our participants. As the PD and PDP groups performed the fMRI task at a similar level, our results are unlikely to be driven by neuropsychological differences. Nevertheless, psychotic symptoms in PD might be associated with a specific neuropsychological profile, and characterizing this is an important goal of future research. A strength of our study is that the PD and PDP groups were well matched in terms of age and current medication: the daily l-DOPA equivalent dose did not differ across the PD groups. Levels of apathy and depression were comparable in the groups. However, a limitation of our study is that we did not record the timings of medication administration on the research day, and so we cannot eliminate the possibility that medication influence at the time of the scan might differ across the PD groups. There was no significant difference in terms of disease duration, although we note that our PDP group had a longer duration of illness than the PD group, in accordance with prior literature showing an association between psychotic symptoms and duration of illness (79–81).

Our findings suggesting a role for striatal and cingulate dysfunction in the pathogenesis of psychosis in PD should be taken in the context of an existing literature investigating PDP, mainly drawn from research modalities other than fMRI. This literature points to cortical abnormalities in PDP, such as reduced gray matter volume in the hippocampus and in frontal, parietal, and occipital cortices (6), and to the presence of Lewy bodies in frontal, cingulate, temporal, and occipital cortices on postmortem examination (9). Psychotic symptoms in PD are often comorbid with dementia and, in some cases, may presage incipient dementia (82). In the related disorder of dementia with Lewy bodies, posterior cortical–thalamic connectivity is related to hallucination severity (83). The dual syndrome hypothesis of cognitive impairments in PD (84) distinguishes between a non-dementia profile of fronto-striatal deficits (manifest in tests of executive function), and a profile of posterior cortical dysfunction with rapidly progressing cognitive decline to dementia. It is possible the psychotic symptoms in PD may emerge through either of these pathological processes or through an interaction of the two. In patients without global cognitive impairment, psychotic symptoms are often comorbid with other non-motor deficits, such as depression and sleep–wakefulness disturbance (3). A full account of psychosis in PD will need to take into account all of these complex factors in explaining why psychotic symptoms are commonly but not universally manifest in PD (12), which pathophysiological mechanisms are unique to PDP, and which are shared with mechanisms of psychotic experiences in schizophrenia or the general population (85). Our study adds to prior evidence of cortical dysfunction in PDP and suggests that there may be factors in common in the pathogenesis of schizophrenia spectrum psychosis and PD psychosis. It is possible that these abnormalities may contribute to the understanding on how monoaminergic function contributes to psychotic symptoms in PD; future pharmacological fMRI studies could specifically investigate this further by comparing, for example, the effects of 5-HT 2a inverse agonists on reward activation in PDP.

## Ethics Statement

The research was approved by the Cambridgeshire 2 NHS Research Ethics Committee. All subjects gave written informed consent in accordance with the Declaration of Helsinki.

## Author Contributions

SG designed the analysis, conducted the analysis, interpreted the results, and wrote the first draft of the paper. AJ designed the data collection, recruited the participants, conducted the data collection, and revised the paper for important intellectual content. GA designed and supervised the analysis and revised the paper for important intellectual content. AE designed the data collection, conducted the data collection, designed the analysis, and revised the paper for important intellectual content. PR designed the data collection, conducted the data collection, and revised the paper for important intellectual content. CT-S designed the analysis, performed analysis, and revised the paper for important intellectual content. TR designed the study, interpreted the results, and revised the manuscript for important intellectual content. RB designed the study, recruited the participants, interpreted the results, and revised the manuscript for important intellectual content. PF designed the study, interpreted the results, and revised the manuscript for important intellectual content. GM had the idea for the study, designed the study, directed the data collection and analysis, drafted the paper, and directed the revisions of the manuscript. All authors gave final approval of the manuscript.

## Conflict of Interest Statement

TR is a consultant for and receives royalties from Cambridge Cognition; is a consultant for and received a research grant from Eli Lilly; received a research grant from GlaxoSmithKline; is a consultant for and received a research grant from Lundbeck; and is a consultant for Teva, Shire Pharmaceuticals, Mundipharma, and Otsuka. PF has consulted for GlaxoSmithKline and Lundbeck and received compensation. The other authors declare that the research was conducted in the absence of any commercial or financial relationships that could be construed as a potential conflict of interest.
