# Supplementary material for: Cortical and Striatal Reward Processing in Parkinson’s Disease Psychosis
Source: Front Neurol. 2017 Apr 24;8:156. doi: 10.3389/fneur.2017.00156 (PMC5402044; doi:10.3389/fneur.2017.00156)
Supplement: Supplementary file 1 [file Image_1.pdf]

Supplementary Image

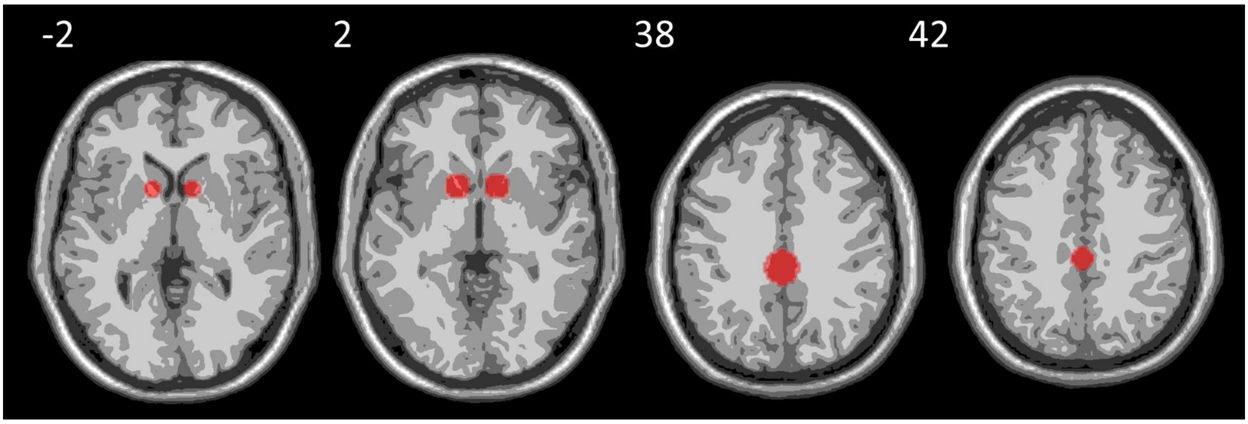

Figure S1. Regions of interest (ROIs)

We focussed on a-priori defined regions of interest (ROIs) comprising medial orbitofrontal cortex, posterior cingulate cortex and bilateral ventral striatum. A single mask from these regions was created using spheres centred on the following MNI coordinates taken from the meta-analysis of reward anticipation by Liu and colleagues (2011): 0, -30, 32 (posterior cingulate, 12mm diameter); 0, 34, -8 (ventromedial prefrontal cortex, 8mm diameter), and bilateral ventral striatum (12, 10, -4, 8mm diameter, and -12, 10, -4, 8mm diameter). Such ROIs are here displayed in red and overlaid on a standard structural image. Left hemisphere is displayed on the left. MNI coordinates are reported.
